# The *LDLR*, *APOB*, and *PCSK9* Variants of Index Patients with Familial Hypercholesterolemia in Russia

**DOI:** 10.3390/genes12010066

**Published:** 2021-01-06

**Authors:** Alexey Meshkov, Alexandra Ershova, Anna Kiseleva, Evgenia Zotova, Evgeniia Sotnikova, Anna Petukhova, Anastasia Zharikova, Pavel Malyshev, Tatyana Rozhkova, Anastasia Blokhina, Alena Limonova, Vasily Ramensky, Mikhail Divashuk, Zukhra Khasanova, Anna Bukaeva, Olga Kurilova, Olga Skirko, Maria Pokrovskaya, Valeriya Mikova, Ekaterina Snigir, Alexsandra Akinshina, Sergey Mitrofanov, Daria Kashtanova, Valentin Makarov, Valeriy Kukharchuk, Sergey Boytsov, Sergey Yudin, Oxana Drapkina

**Affiliations:** 1 National Medical Research Center for Therapy and Preventive Medicine, Petroverigsky per., 10, bld. 3, 101000 Moscow, Russia; alersh@mail.ru (A.E.); sanyutabe@gmail.com (A.K.); sotnikova.evgeniya@gmail.com (E.S.); azharikova89@gmail.com (A.Z.); franny349@gmail.com (A.B.); limonova-alena@yandex.ru (A.L.); ramensky@gmail.com (V.R.); divashuk@gmail.com (M.D.); olga_kurilova81@mail.ru (O.K.); ops_70@mail.ru (O.S.); MPokrovskaya@gnicpm.ru (M.P.); drapkina@bk.ru (O.D.); 2 Centre for Strategic Planning of FMBA of Russia, Pogodinskaya Street, 10, bld. 1, 119121 Moscow, Russia; EZotova@cspmz.ru (E.Z.); APetukhova@cspmz.ru (A.P.); annbukaeva@gmail.com (A.B.); VMikova@cspmz.ru (V.M.); ESnigir@cspmz.ru (E.S.); Akinshina@cspmz.ru (A.A.); mitrofanov@cspmz.ru (S.M.); DKashtanova@cspmz.ru (D.K.); makarov@cspmz.ru (V.M.); yudin@cspmz.ru (S.Y.); 3 Faculty of Bioengineering and Bioinformatics, Lomonosov Moscow State University, Leninskie Gory, 1-73, 119991 Moscow, Russia; 4 National Medical Research Center for Cardiology, 3-ya Cherepkovskaya Street, 15A, 121552 Moscow, Russia; pavel-malyshev@mail.ru (P.M.); rozhkova.ta@mail.ru (T.R.); zukhra@yandex.ru (Z.K.); v_kukharch@mail.ru (V.K.); prof.boytsov@gmail.com (S.B.)

**Keywords:** familial hypercholesterolemia, Russian, whole genome sequencing, *LDLR*, *APOB*, *PCSK9*

## Abstract

Familial hypercholesterolemia (FH) is a common autosomal codominant disorder, characterized by elevated low-density lipoprotein cholesterol levels causing premature atherosclerotic cardiovascular disease. About 2900 variants of *LDLR*, *APOB*, and *PCSK9* genes potentially associated with FH have been described earlier. Nevertheless, the genetics of FH in a Russian population is poorly understood. The aim of this study is to present data on the spectrum of *LDLR*, *APOB*, and *PCSK9* gene variants in a cohort of 595 index Russian patients with FH, as well as an additional systematic analysis of the literature for the period of 1995–2020 on *LDLR*, *APOB* and *PCSK9* gene variants described in Russian patients with FH. We used targeted and whole genome sequencing to search for variants. Accordingly, when combining our novel data and the data of a systematic literature review, we described 224 variants: 187 variants in *LDLR*, 14 variants in *APOB,* and 23 variants in *PCSK9*. A significant proportion of variants, 81 of 224 (36.1%), were not described earlier in FH patients in other populations and may be specific for Russia. Thus, this study significantly supplements knowledge about the spectrum of variants causing FH in Russia and may contribute to a wider implementation of genetic diagnostics in FH patients in Russia.

## 1. Introduction

Familial hypercholesterolemia (FH) is a common autosomal codominant disorder, characterized by elevated low-density lipoprotein (LDL) cholesterol levels causing premature atherosclerotic cardiovascular disease [1]. In two meta-analyses of 2020, similar results were obtained on the prevalence of heterozygous FH (HeFH) in the general population: one in 311 and one in 313, respectively [2,3]. The prevalence of homozygous FH (HoFH) is one in 300,000 [4]. Mutations in one of the three genes (low-density lipoprotein receptor gene (*LDLR*), apolipoprotein B gene (*APOB*) and proprotein convertase subtilisin/kexin type 9 gene (*PCSK9*)) cause both HeFH and HoFH, and these genes account for the vast majority of genetically confirmed cases of FH [1]. For *LDLRAP1*, *LIPA*, *ABCG5* and *ABCG8* genes, two mutant alleles act recessively, producing a severe phenotype consistent with HoFH, but only single families have been described [1]. About 2900 variants in the *LDLR*, *APOB* and *PCSK9* genes potentially associated with FH have been described by the members of the ClinGen FH Variant Curation Expert Panel from 13 different countries [5]. Nevertheless, the genetics of FH in a Russian population is still poorly understood, with only about 60 variants of *LDLR* and *APOB* genes described in single publications [6,7,8,9,10]. The aim of this study is to present data on the spectrum of the *LDLR*, *APOB* and *PCSK9* gene variants in a cohort of 595 index Russian patients with FH, and to perform an additional systematic analysis of the literature for the period of 1995–2020 on *LDLR*, *APOB* and *PCSK9* gene variants described in Russian FH patients. 

## 2. Materials and Methods 

### 2.1. Clinical Description of the Patients

The study included index patients (*n* = 595) with clinically and genetically confirmed diagnosis of HeFH or HoFH examined by researchers at the National Medical Research Center for Therapy and Preventive Medicine (Moscow, Russia) and the National Medical Research Center for Cardiology (Moscow, Russia). HeFH was determined using the Dutch Lipid Clinical Network Criteria (DLCN) including the results of genetic testing [11]. This diagnosis was established when the DLCN score was six points or more. The diagnosis of HoFH was determined using the guidance of the European Atherosclerosis Society [4]. Blood for genetic analysis was stored in the Biobank of the National Medical Research Center for Therapy and Preventive Medicine (Moscow, Russia). Targeted sequencing and Sanger sequencing were performed at the National Medical Research Center for Therapy and Preventive Medicine (Moscow, Russia). Whole genome sequencing was performed at the Center for Strategic Planning of the Federal Medical Biological Agency (Moscow, Russia). This study was performed in accordance with the Declaration of Helsinki and was approved by the Committee on the Ethics issues in clinical cardiology of the National Medical Research Center for Cardiology (Moscow, Russia) and by the Institutional Review Boards of the National Research Center for Therapy and Preventive Medicine (Moscow, Russia) with written informed consent obtained from each participant and/or their legal representative, as appropriate. 

### 2.2. Systematic Review

We performed a systematic review of all relevant peer-reviewed published articles involving patients with FH from Russia. The search strategy was designed to cover all articles published in English using three literature databases (Scopus, Web of Science and PubMed) from 1995 to July 2020. The search terms were: (“Familial hypercholesterolemia” OR “LDLR” OR “APOB” OR “PCSK9”) and (“Russia” OR “Russian”). The eligible articles were screened for both the titles and abstracts.

### 2.3. Molecular Genetic Analysis

#### 2.3.1. Target Sequencing

DNA was isolated using the QIAamp DNA Blood Mini Kit (Qiagen, Hilden, Germany). DNA concentration was assessed with a Qubit 4.0 fluorimeter (Thermo Fisher Scientific, Waltham, MA, USA). Target sequencing was performed with two platforms: Ion S5 (Thermo Fisher Scientific, Waltham, MA, USA) and Nextseq550 (Illumina, San Diego, CA, USA). For sequencing on Ion S5, DNA libraries were prepared on an Ion Chef System (Thermo Fisher Scientific, Waltham, MA, USA) using a custom panel designed automatically by Ion AmpliSeq Designer software v7.4.2 (Thermo Fisher Scientific, Waltham, MA, USA). The panel flanked exonic and adjacent intronic sequences of 25 genes (UTR + CDS + 100 bp padding). VCF files were generated from BAM files on a Torrent Server (Thermo Fisher Scientific, Waltham, MA, USA) with default parameters. VCF files were annotated using Ion Reporter (Thermo Fisher Scientific, Waltham, MA, USA) with Annotate Variants analysis tool. For Nextseq 550, the library preparation was performed using the SeqCap EZ Prime Choice Library kit (Roche, Basel, Switzerland). Two Roche panels were used, consisting of 24 (CDS + 25 bp padding) and 244 (CDS + 25 bp padding) genes. All three panels included the *LDLR*, *APOB* and *PCSK9* genes. All stages of sequencing were carried out according to the manufacturers’ protocols. Reads were aligned to the reference genome (GRCh37). Sequencing analysis resulted in fastq files. Data processing was performed with BWA, Picard, bcftools, GATK3 and generally followed the GATK best practices for variant calling. We applied standard GATK hard filters for single nucleotide substitutions (MQ, QD, FS, SOR, MQRankSum, QUAL, ReadPosRankSum) and for short insertions and deletions (QD, FS, QUAL, ReadPosRankSum). Single nucleotide variants and short indels were annotated with ANNOVAR.

#### 2.3.2. Whole Genome Sequencing and Bioinformatic Analysis

DNA was extracted from whole blood sample using QIAamp^®^ DNA Mini Kit (Qiagen, Hilden, Germany). A WGS library was prepared using Nextera DNA Flex kit (Illumina, San Diego, CA, USA) according to manufacturer instructions. Paired-end sequencing (150 bp) was performed to mean sequencing coverage of 30× or more. Reads were aligned to the reference genome (GRCh38) and small variants were called using Dragen Bio-IT platform (Illumina, San Diego, CA, USA) and joint-called with GLnexus [12]. 

**Figure 1:**
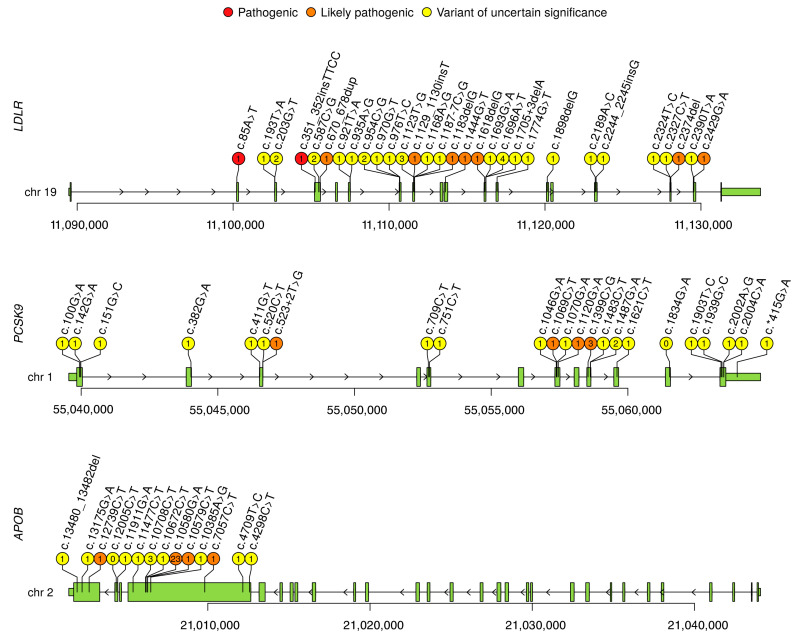
Variants in *LDLR*, *PSCK9,* and *APOB* genes, specific for the Russian population. For the *LDLR* gene, due to the large quantity, only 30 novel variants found in this study are shown (with the exception of four large structural variants presented in Figure 2). Number of index patient is indicated in the circle (0 is for variants found in other studies), color indicates clinical interpretation: red, orange and yellow for pathogenic (P), likely pathogenic (LP) and variant of uncertain significance (VUS), respectively. Coordinates are given in hg38 assembly.

Structural variant (SV) calling was performed with smoove software [13]. Annotation was performed using an Ensembl Variant Effect Predictor (VEP) [14]. All variants were visually inspected in an Integrative Genomics Viewer (IGV) [15] and breakpoint regions were investigated with PCR and Sanger sequencing. Mobile elements (ME) SVA, LINE1 and Alu were called using MELT software [16] and annotated with VEP [14]. Images were prepared using the R programming language. For Figure 1 a trackViewer package was used [17].

#### 2.3.3. Clinical Interpretation

The following canonical transcripts were used in this work: NM_000527.5 (*LDLR*), NM_000384.3 (*APOB*), and NM_174936.4 *(PCSK9*). For clinical interpretation, short genetic variants with overall frequencies for European (non-Finnish) in the gnomAD database of <0.5%, or missing in the gnomAD, were selected. SV-only variants with frequencies of <0.5% for European (non-Finnish) were left for evaluation. No ME insertions were found for *LDLR*, *APOB* or *PSCK9*. Evaluation of the pathogenicity of the variants was carried out in accordance with the recommendations of the American College of Medical Genetics and Genomics (ACMG) with modifications [18]. The following types of variants are reported in the article: pathogenic (P), likely pathogenic (LP) and variant of unknown significance (VUS). All variants were analyzed for their presence in the databases (LOVD, ClinVar and HGMD) [5,19].

#### 2.3.4. Sanger Sequencing

The validation of NGS results was done by Sanger sequencing. PCRs were performed in 20 μL of a mixture containing 0.2 mM of each nucleotide, 1× PCR buffer, 20 ng of the DNA, 10 ng of each primer, 2.5 U of DNA polymerase. Amplification was performed on a GeneAmp PCR System 9700 thermocycler (Thermo Fisher Scientific, Waltham, MA, USA) with the following parameters: 95 °C—300 s; 30 cycles: 95 °C—30 s, 62 °C—30 s, 72 °C—30 s; 72 °C—600 s. Before the Sanger reaction, the obtained amplicons were purified using ExoSAP-IT (Affymetrix, Santa Clara, CA, USA) according to the manufacturer’s protocol. The nucleotide sequence of PCR products was determined using the ABI PRISM^®^ BigDye™ Terminator reagent kit v. 3.1 followed by analysis of the reaction products on an automated DNA sequencer Applied Biosystem 3500 DNA Analyzer (Thermo Fisher Scientific, Waltham, MA, USA). 

## 3. Results

### 3.1. Systematic Literature Review

The search strategy described above yielded 665 citations; 474 remained after duplicate removal. After the analysis of the abstracts referring to genetic testing or *LDLR*, *APOB* and *PCSK9* variants in FH patients, 27 articles were selected, of which 25 contained data on the *LDLR*, *APOB*, and *PCSK9* variants, including three of previously published articles by our group [6,7,8,9,10,20,21,22,23,24,25,26,27,28,29,30,31,32,33,34,35,36,37,38,39]. These articles describe 91 causal variants of *LDLR* gene, one variant of *APOB,* and one variant of *PCSK9* (Figure 1, Table A1, Table A2 and Table A3 in Appendix A).

### 3.2. Genetic Test Results

**Figure 2:**
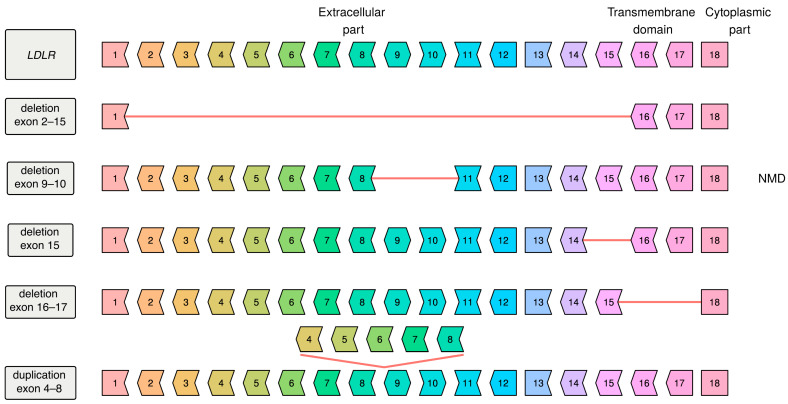
Exonic structure of the native *LDLR* gene and its large structural variants found in this study. Exon border shape (flat and right or left pointing) shows the phase of the reading frame (+0; +1; +2); if borders don’t match, a frame shift occurs (deletions exon 9–10 and 16–17). NMD marks a variant that likely leads to the nonsense-mediated decay.

In our study we performed genetic testing of 595 unrelated patients with FH, of which six patients demonstrated the phenotype of HoFH and the rest had clinical features of HeFH. Target sequencing was performed for 401 patients and whole genome sequencing was performed for 405 patients (both methods were performed for 211 patients). In 405 WGS patients we called SNPs, short indels, long SVs and ME insertions. We identified 122 different potentially causative variants in *LDLR*, 13 variants in *APOB*, and 21 variants in *PCSK9* in 294 unrelated patients (Figure 1 and Figure 2, Table A1, Table A2 and Table A3). No potentially causative variants were found in 301 of 595 patients (50.6%). Out of these 294 patients, one patient was a true homozygote, four compound heterozygotes with two *LDLR* variants on different chromosomes (in trans), one compound heterozygote with two *LDLR* variants on the same chromosome (in cis), two compound heterozygotes with two *LDLR* variants of unknown mutual arrangement of alleles, six double heterozygotes (harboring two variants in two different genes) and one patient with three variants in three genes (Table A4), the rest were simple heterozygotes. A total of 34 variants in *LDLR*, six variants in *APOB* and six variants in *PCSK9*, were found in this study for the first time. Most of these variants were unique but some *LDLR* variants occurred in several unrelated patients: p.Cys68Phe, p.Pro196Arg, p.Cys318Trp, p.Tyr375Asp and p.Ile566Phe. Of 35 variants previously described in the literature [6,7,8,9,10,20,21,22,23,24,25,26,27,28,29,30,31,32,33,34,35,36,37,38,39] only for the Russian population, six variants were also found in this study. Most of these variants were also unique, except for variant *LDLR*-p.Cys160Gly, that was found in six unrelated patients. Of all variants (the percentage of all identified potentially causative alleles (310 alleles found in this study)) the most common were: *LDLR*-p.Gly592Glu—9.4%, *LDLR*-p.Leu401His—9%, *APOB*-p.Arg3527Gln—7.4%, *LDLR*-p.Cys329Tyr—2.6%, *LDLR*-p.Cys160Gly—1.9%. Most of the variants described above were SNPs and short indels. Only five large SVs were found in this study and all of them in *LDLR* gene (Figure 2). Four novel deletions were found and a tandem duplication previously described in a patient of Czech origin (ClinVar ID: 251140). No ME insertions were found in any of the studied genes.

### 3.3. Description of All Variants in Russia

In total, when combining our data (156 *LDLR*, *APOB* and *PCSK9* variants) and the data of the systematic review (91 *LDLR*, *APOB* and *PCSK9* variants), we described 224 variants: 187 *LDLR* variants, 14 *APOB* variants, and 23 *PCSK9* variants (Table A1, Table A2 and Table A3). A significant proportion of variants—36.1% (67 *LDLR* variants, six *APOB* variants and eight *PCSK9* variants)— was not described in FH patients in other populations and may be specific for Russia. 

In accordance with the criteria of pathogenicity, 38 *LDLR* variants were classified as pathogenic (P), 53 as likely pathogenic (LP) and 95 as variant of unknown significance (VUS). In the *APOB* gene there were four LP and 10 VUS, and in the *PCSK9* gene four LP and 19 VUS (Table 1).

## 4. Discussion

This study was based on the largest number of participants of any genetic FH study in Russia to date. Including collected literature data, this study reported 224 variants found in the Russian population, either novel or reported before, with 81 variants described only in Russian FH patients. These data on the spectrum of the *LDLR*, *APOB* and *PCSK9* variants can be useful for clinical interpretation when carrying out a genetic diagnosis of FH in Russia. It also improved knowledge about the genetics of FH in general. Thus, according the results of this study, Russia is ranked fourth among countries with the largest number of variants described in FH patients, after the United Kingdom, the Netherlands and Italy [18]. In our study, we did not carry out a functional analysis of the identified variants and used ACMG recommendations to assess their pathogenicity. About half of the variants described here were assigned a category of uncertain significance and, possibly, in the future with the advent of new data, their causality may be revised. It would also be desirable to assess the clinical significance of the combined effect of two or more variants identified in patients with HeFH (Table A4).

The WGS-based SV analysis was performed for 405 patients for whom no relevant variants were found by targeted sequencing. The fact that no large SVs were found either in *PCSK9* or in *APOB* may be explained by their gain-of-function pathogenicity model. Taking into account the literature data, nine large rearrangements in *LDLR* were described for the Russian patients earlier and their proportion of the total number of unique variants (*n* = 187) of the *LDLR* gene was 4.8%, which is slightly less than the share of large *LDLR* rearrangements in the ClinVar database (6.1%) [5]. The presence of large deletions, encompassing exonic *LDLR* regions, suggests that multiplex ligation-dependent probe amplification could be a useful method in genetic confirmation of FH.

## 5. Conclusions

This study significantly supplements knowledge about the spectrum of variants causing FH in Russia and may contribute to a wider implementation of genetic diagnostics in Russian FH patients.

## Figures and Tables

**Table 1:** Variants, found in this study.

Gene	Total (P/LP/VUS)	Possibly Unique including Novel for the Russian Population and Described Earlier (P/LP/VUS)	Novel (P/LP/VUS)	Described in Other World Populations
*LDLR*	187 (38/53/95) *	67 (11/19/37)	34 (3/10/21)	120 (27/34/58)
*APOB*	14 (0/4/10)	6 (0/1/5)	6(0/1/5)	8 (0/3/5)
*PCSK9*	23 (0/4/19)	8(0/1/7)	6 (0/1/5)	5 (0/3/12)

Novel: variants found in this study for the first time. Possibly unique for the Russian population: variants found in this study and previously described only for the Russian population. (*)—for one variant it was impossible to determine the category of pathogenicity. However, it was earlier described in the literature as pathogenic.

## Data Availability

The datasets used and/or analyzed during the current study are available from the corresponding author on reasonable request.

## Appendix A

**Table A1:** List of the *LDLR* variants described in Russian patients.

Number of Index Patients ^1^	Variant Data ^2^	Exon	DNA Change	Protein Change	dbSNP ID	gnomAD MAF (v. 2.1.1)	ACMG Interpretation	ClinVar Interpretation	ClinVar ID	References
1	1	1i–15i	c.68-366_2312-791del				P			
0	6	2		p.Cys27Trp	rs2228671		VUS	P/LP	226304	[30]
1	1	2	c.85A > T	p.Arg29Ter	rs879254401		P	P	251011	
0	6	2	c.97C > T	p.Gln33Ter	rs121908024	0.000007963	P	P	3683	[6,20,30]
0	6	3	c.191_313del	p.Leu64_Pro105delinsSer			P			[9]
0	5	3	c.193_202delTCTGTCACCTinsGGACTTCA	p.Ser65Glyfs * 64			LP			[8,10,25,27,29]
1	1	3	c.193T > A	p.Ser65Thr			VUS			
0	5	3	c.195dupT	p.Val66Cysfs * 64	rs879254435		P	P	251075	[8,10,25,29]
2	4	3	c.200C > T	p.Thr67Ile	rs1337448484	0.00001060	VUS	VUS	629411	
2	1	3	c.203G > T	p.Cys68Phe			VUS			
2	2	3	c.230dup	p.Arg78ProfsTer55	rs879254440		P	P	251083	[30,37,39]
1	4	3	c.241C > T	p.Arg81Cys	rs730882078	0.000007953	VUS	P/LP/VUS	183083	
0	6	3	c.245G > C	p.Cys82Ser			VUS	VUS	431509	[10]
1	4	3	c.246C > A	p.Cys82Ter	rs875989891		P	P	226309	
0	6	3	c.285C > A	p.Cys95Ter	rs139400379		P	P	251115	[20,21,30]
0	6	3i	c.313 + 1G > A		rs112029328	0.00002784	P	P/LP	3736	[6,20]
0	5	3i	c.313 + 2T > G				LP			[10]
1	3	4–8	c.317-1185dup	p.Pro106_Val395dup			LP			[9]
2	4	4	c.326G > A	p.Cys109Tyr	rs121908042	0.000003996	LP	P/LP	226319	
2	4	4	c.343C > T	p.Arg115Cys	rs774723292	0.00002792	LP	P/LP/VUS	251162	
1	4	4	c.347G > A	p.Cys116Tyr			LP			
0	6	4	c.347_349delGCC	p.Cys116_His117delinsTyr	rs879254483		LP	LP	251164	[20,26,30]
1	1	4	c.351_352insTTCC	p.Asp118PhefsTer13			P			[7]
1	5	4	c.355_356insTTCC	p.Gly119ValfsTer12			P			[9]
1	4	4	c.420G > C	p.Glu140Asp	rs879254520		LP	P/LP	251216	
0	5	4	c.444T > G	p.Cys148Trp	rs879254528		LP	LP	251228	[20,24,30]
0	6	4	c.451G > C	p.Ala151Pro	rs763233960	0.00001195	LP	VUS	251234	[20,28,30]
6	2	4	c.478T > G	p.Cys160Gly	rs879254540		LP	LP	251248	[20,24,29,30]
0	6	4	c.499T > C	p.Cys167Arg	rs879254547		LP	P/LP	251255	[20,28]
1	4	4	c.502G > C	p.Asp168His	rs200727689		LP	P/LP	251258	
1	4	4	c.519C > G	p.Cys173Trp	rs769318035	0.000007958	LP	P/LP	251277	
0	6	4	c.530C > T	p.Ser177Leu	rs121908026	0.00001592	LP	P/LP	3686	[9]
1	3	4	c.534T > G	p.Asp178Glu	rs879254566		LP	P/LP	251287	[36]
0	6	4	c.542C > T	p.Pro181Leu	rs557344672	0.000007958	LP	LP	431512	[9]
2	4	4	c.551G > A	p.Cys184Tyr	rs121908039	0.00009554	LP	P/LP	3739	
2	1	4	c.587C > G	p.Pro196Arg			VUS			
0	6	4	c.618T > G	p.Ser206Arg	rs879254595		LP	P/LP	251325	[8,10,25,29]
5	3	4	c.622G > A	p.Glu208Lys	rs879254597		LP	P/LP	251328	[32]
0	6	4	c.626G > A	p.Cys209Tyr	rs879254600		LP	P/LP	251332	[20,28,30]
5	3	4	c.654_656delTGG	p.Gly219del	rs121908027		P	P/LP	226329	[6,20,23,29,30,31]
1	3	4	c.658_663delCCCGAC	p.Pro220_Asp221del	rs1555803409		LP	P	440589	[9]
0	5	4–6	Del 5 kb incl. ex. 4–6				LP			[38]
3	4	4	c.666C > A	p.Cys222Ter	rs756613387	0.000004005	P	P	251364	
1	4	4	c.672_686delCAAATCTGACGAGGA	p.Asp224_Glu228del	rs1555803439		LP	LP	441189	[7]
0	5	4	c.670_671insG	p.Asp224GlyfsTer4	rs879254629		P	P	251372	[6,20,30]
1	1	4	c.670_678dup	p.Asp224_Ser226dup			LP			[7]
3	4	4	c.682G > A	p.Glu228Lys	rs121908029	0.00001614	LP	P/LP	3691	[30,37,39]
0	6	4	c.682G > T	p.Glu228Ter	rs121908029	0.00001074	P	P/LP	226333	[6,20,30]
1	4	4	c.693C > A	p.Cys231Ter	rs121908035		P	P/LP	3730	
0	5	5		p.Glu240Ter *			LP			[30]
0	6	5		p.Glu240Lys *			VUS	P/LP/VUS	200920	[30]
1	4	5	c.768C > A	p.Asp256Glu	rs879254671		VUS	LP	438322	
0	6	5		p.Cys261Phe *			VUS	LP	3740	[30]
0	6	5	c.796G > A	p.Asp266Asn	rs875989907	0.00001193	LP	P/LP	226334	[34,36]
3	4	5	c.798T > A	p.Asp266Glu	rs139043155	0.00003535	VUS	P/LP/VUS	161287	
2	3	5	c.810C > A	p.Cys270Ter	rs773328511		P	P	251465	[6,20,30]
1	4	6	c.825_826delCT	p.Cys276ArgfsTer24	rs879254691		P	P	251478	
2	4	6	c.829G > A	p.Glu277Lys	rs148698650	0.0005056	VUS/LB	P/VUS/LB/B	183097	
0	6	6		p.Glu288Lys			VUS	P/LP/VUS	161268	[30]
2	4	6	c.905G > T	p.Cys302Phe	rs879254715		LP	P	430768	
0	6	6	c.922G > A	p.Glu308Lys	rs879254721		VUS	LP	251528	[9]
0	6	6	c.925_931delCCCATCA	p.Pro309LysfsTer59	rs387906304		P	P	3729	[6,8,10,20,22,25,29,30]
1	1	6	c.921T > A	p.Asp307Glu			VUS			
1	1	6	c.935A > G	p.Glu312Gly	rs1380197577	0.000003984	VUS			
0	5	6	c.939_940 + 3delCGGTG	p.Cys313AspfsTer17	rs879254727		P	P	251536	[6,20,30]
3	3	6i	c.940 + 3_940 + 6del				VUS	P/VUS	869390	[9]
0	5	5i_6i	c.817 + 303_940 + 943del	p.Val273_Cys313del			VUS			[32]
1	4	6i	c.941-3C > G				VUS			
1	4	6i	c.941-2A > G		rs112366278		P	P/LP	251554	
1	4	7	c.949G > A	p.Glu317Lys	rs746834464	0.00005311	VUS	P/LP	251567	
2	1	7	c.954C > G	p.Cys318Trp			VUS			
1	1	7	c.970G > T	p.Gly324Cys			VUS			
1	4	7	c.974G > A	p.Cys325Tyr	rs879254746		VUS	P/LP	251580	
1	1	7	c.976T > C	p.Ser326Pro			VUS			
8	3	7	c.986G > A	p.Cys329Tyr	rs761954844	0.00002479	VUS	P/LP/LB	226344	[6,9,20,30,36]
0	6	7	c.1009G > A	p.Glu337Lys	rs539080792	0.0000935	VUS	VUS	523729	[34,36]
0	6	7			rs755757866	NA (G > A)\0.000007967 (G > T)		LP (G > A)\NA (G > T)	251600 (G > A)\NA (G > T)	[34,36]
1	4	7	c.1027G > A	p.Gly343Ser	rs730882096	0.00002832	VUS	P/LP/VUS	183106	
1	4	7	c.1048C > T	p.Arg350Ter	rs769737896	0.000007977	P	P	226342	
1	3	7	c.1054T > C	p.Cys352Arg	rs879254769		VUS	LP	251618	[9,34,36]
3	4	7i	c.1061-8T > C		rs72658861	0.005498	VUS/LB	VUS/PB/B	36451	
3	1	8	c.1123T > G	p.Tyr375Asp			VUS			
1	1	8	c.1129_1130insT	p.Cys377LeufsTer1			LP			
1	4	8	c.1162del	p.His388ThrfsTer25			P	P	226348	
1	1	8	c.1168A > G	p.Lys390Glu			VUS			
1	1	8	c.1183delG	p.V395fs			LP			
1	4	8i	c.1186 + 1G > T		rs730880131		P	P	180403	
1	1	8i-10i	c.1186 + 568_1586 + 1067del				LP			
3	4	8i	c.1187-10G > A		rs765696008	0.00002798	VUS	P/LP	226349	
1	1	8i	c.1187-7C > G				VUS			
28	3	9	c.1202T > A	p.Leu401His	rs121908038		VUS	P/LP	3735	[6,20,34,36]
2	4	9	c.1217G > A	p.Arg406Gln	rs552422789	0.00001593	VUS	P/LP/VUS	228798	
5	3	9	c.1222G > A	p.Glu408Lys	rs137943601	0.000007965	LP	P/LP	36453	[10]
0	5	9		p.Arg410Gly *			VUS			[30]
0	5	9		p.Met412Val *			VUS			[30]
4	3	9	c.1246C > T	p.Arg416Trp	rs570942190	0.00002389	LP	P/LP	183110	[9,30,34,36]
1	3	9	c.1252G > T	p.Glu418Ter	rs869320651		P	P	251755	[20,26,30]
0	5	9		p.Glu418Gly *			VUS			[30]
0	6	9	c.1277T > C	p.Leu426Pro	rs879254851		VUS	P/LB	251763	[25]
1	4	9	c.1285G > A	p.Val429Met	rs28942078	0.00001194	LP	P/LP	3694	
0	5	9	c.1291_1331del41	p.Ala431Ter	rs879254854		LP			[6,20,30]
1	4	9	c.1292C > T	p.Ala431Val			VUS			
0	6	9		p.Leu432Arg *			VUS			[30]
0	5	9		p.Asp433Glu *	rs778309692	0.000003980	VUS			[30]
0	6	9		p.Asp433His *			VUS			[30]
0	6	9		p.Asp433Tyr *			VUS			[30]
0	6	9	c.1302delG	p.Glu435MetfsTer15			P			[6,20,30]
1	4	9	c.1322T > A	p.Ile441Asn	rs879254862		VUS	LP	251782	
2	2	9	c.1327T > C	p.Trp443Arg	rs773566855	0.000003980	LP			[9,10,30,33]
0	6	9	c.1328G > A	p.Trp443Ter	rs879254866		P	P	251789	[6,20]
0	6	9	c.1340C > G	p.Ser447Cys	rs879254870		VUS	LP	251797	[8,10,25]
0	6	9i	c.1358 + 1G > A		rs775924858		P	P/LP	251802	[6,20]
1	1	10	c.1444G > T	p.Asp482Tyr	rs139624145		LP	LP	251845	[30]
1	2	10	c.1465T > A	p.Tyr489Asn			VUS			[9]
1	4	10	c.1471A > G	p.Thr491Ala			VUS			
1	4	10	c.1474G > A	p.Asp492Asn	rs373646964	0.00002386	VUS	P/LP/VUS	161285	
1	4	10	c.1502C > T	p.Ala501Val	rs755667663	0.000007954	LP	P/LP	251874	
0	6	10	c.1532T > C	p.Leu511Ser	rs879254932		VUS	LP	251887	[8,25,33]
1	4	10	c.1561G > A	p.Ala521Thr	rs879254940		VUS	VUS	251898	
1	4	10	c.1577C > A	p.Pro526His	rs879254944		VUS	VUS	496019	
1	4	10i	c.1586 + 5G > A		rs781362878	0.00003189	VUS	LP/VUS	251909	
1	1	11	c.1618delG	p.Ala540ProfsTer8			LP			
1	3	11	c.1633G > A	p.Gly545Arg	rs879254965		LP	P/LP	251942	[9]
0	6	11		p.Gly549Asp *	rs28941776	0.00002386	VUS	P/LP	3698	[30]
0	5	11	c.1655_1672del				LP			[34,36]
1	4	11	c.1672G > T	p.Glu558Ter	rs879254980		P	P	251964	
0	5	11		p.Glu558Lys *			VUS			[30]
0	5	11	c.1686_1693delGCCCAATGinsT	p.Trp562CysfsTer5	rs879254984		P	P	251968	[8,25,33]
1	1	11	c.1693G > A	p.Gly565Ser	rs1344561983	0.000003978	VUS			
4	1	11	c.1696A > T	p.Ile566Phe			VUS			
1	1	11	c.1705 + 3delA				VUS			
0	6	11		p.Leu568Va l *			VUS			[30]
1	4	12	c.1706-10G > A		rs17248882	0.002220	VUS/LB	VUS/LB/B	226368	
1	4	12	c.1708_1710delCTC	p.Leu571del	rs772492150	0.000007953	VUS			
1	4	12	c.1729T > C	p.Trp577Arg	rs879255000		LP	P/LP	252001	
0	5	12	c.1741A > C	p.Lys581Gln			VUS			[9]
2	4	12	c.1747C > T	p.His583Tyr	rs730882109	0.0001025	VUS	P/LP	200921	
1	4	12	c.1750T > C	p.Ser584Pro	rs879255010		VUS	LP/VUS	252015	
2	2	12	c.1756T > C	p.Ser586Pro			VUS			[9]
1	1	12	c.1774G > T	p.Gly592Trp			VUS			
29	3	12	c.1775G > A	p.Gly592Glu	rs137929307	0.00005656	LP	P/LP	161271	[9,20,21,30]
2	4	12	c.1784G > A	p.Arg595Gln	rs201102492	0.00003889	VUS	P/LP/VUS	183126	
0	5	12		p.Leu605Val *			VUS			[30]
0	5	12		p.Leu605Arg *			VUS			[30]
0	5	12		p.Ala612Gly *			VUS			[30]
1	2	12i	c.1846-3T > G				VUS			[9]
0	5	13	c.1855–1856insA	p.Phe619TyrfsTer26	rs879255053		P	P	252082	[6,20,30]
0	6	13	c.1859G > C	p.Trp620Ser			VUS			[33]
1	3	13	c.1864G > A	p.Asp622Asn	rs879255059		LP	LP	252092	[6,20,30]
1	1	13	c.1898delG	p.Arg633fs			VUS			
2	4	13	c.1898G > A	p.Arg633His	rs754536745	0.00002121	VUS	P/LP/VUS	226380	
0	5	13	c.1936C > A	p.Leu646Ile	rs779940524	0.000003977	VUS	LP	252118	[8,10,25]
1	4	13	c.1945C > T	p.Pro649Ser	rs879255080		VUS	LP	252121	
3	4	13	c.1955T > C	p.Met652Thr	rs875989936	0.000003977	VUS	LP/VUS	226382	
1	4	13	c.1966C > A	p.His656Asn	rs762815611		LP	B/LP/VUS	252136	
1	4	14	c.1998G > A	p.Trp666Ter	rs752935814		P	P	252161	
0	5	14	c.1999T > A	p.Cys667Ser	rs150021927		VUS	LP	252162	[6,20,30]
1	4	14	c.2001_2002delTG	p.Cys667_Glu668delinsTer	rs1600743301		LP	P	630543	
1	4	14	c.2043C > A	p.Cys681Ter	rs121908031	0.000007959	P	P/LP	3699	
3	4	14	c.2089G > C	p.Ala697Pro	rs776217028		VUS	LP	252213	
0	6	14i–16i	c.2141-966_2390-330del	p.Glu714_Ile796del			LP			[9]
1	1	14i-15i	c.2141-799_2311 + 689del				LP			
1	1	15	c.2189A > C	p.Lys730Thr			VUS			
0	5	15	c.2191delG	p.Val731SerfsTer6	rs879255161		P	P	252253	[8,25,29,33]
0	6	15	c.2215C > T	p.Gln739Ter	rs370018159		P	P/LP	252258	[9]
1	4	15	c.2230C > T	p.Arg744Ter	rs200793488	0.000003979	LP	P	430795	
0	6	15	c.2231G > A	p.Arg744Gln	rs137853963	0.0008030	VUS	LP/VUS/LB/B	68104	[20,21]
1	1	15	c.2244_2245insG	p.Thr749AspfsTer33			VUS			
1	1	15i-17i	c.2312-2107_2547 + 620del				LP			
1	1	16	c.2324T > C	p.Val775Ala	rs780300776	0.00002121	VUS	LB/VUS	440691	
0	5	16	c.2326G > T	p.Ala776Ser			VUS			[32]
1	1	16	c.2327C > T	p.Ala776Val			VUS			
1	4	16	c.2347A > C	p.Lys783Gln	rs769318961	0.000007954	VUS			
1	1	16	c.2374del	p.Ile792LeufsTer137			LP			
3	3	16	c.2389G > A	p.Val797Met	rs750518671	0.000007957	VUS	P/LP/VUS	226393	[6,20,30]
1	4	16	c.2389G > C	p.Val797Leu	rs750518671		VUS	VUS	565983	
1	4	16	c.2389 + 2T > G		rs879255188		P	LP	252302	
4	3	16i	c.2389 + 5G > C		rs879255191		VUS	VUS	661713	[9]
2	4	16	c.2389 + 5G > A		rs879255191		VUS	P/LB	252306	
1	1	17	c.2390T > A	p.Val797Glu			VUS			
2	4	17	c.2416_2417insG	p.Val806fs			P	P/LP	252330	
0	6	17	c.2416dupG	p.Val806GlyfsTer11	rs773618064		P	P/LP	252330	[9]
1	1	17	c.2429G > A	p.Trp810Ter			LP			
1	4	17	c.2448G > C	p.Lys816Asn	rs1399689294	0.00003186	VUS	LP/VUS	440698	
1	4	17	c.2473A > G	p.Asn825Asp	rs879255215		VUS	LP	252340	
1	3	17	c.2479G > A	p.Val827Ile	rs137853964	0.0009193	VUS/LB	LP/VUS/LB/B	36462	[6,20,34,36]
1	4	17	c.2531G > A	p.Gly844Asp	rs121908037		VUS	LP	3734	

^1^ Only for variants found in this study a number of index patients is given. Variants from systematic review are labelled with “0”. ^2^ 1–described only in this study, 2–described in this study and in other studies in Russia, 3–described in this study, in other studies in Russia and other countries, 4–described in this study and other countries, 5–did not occur in this study, described in other studies in Russia, 6–did not occur in this study, described in other studies in Russia and other countries. * No data on coding sequence alteration in reference.

**Table A2:** List of the *PSCK9* variants described in Russian patients.

Number of Index Patients ^1^	Exon	DNA Change	Protein Change	dbSNP ID	gnomAD MAF (v. 2.1.1)	ACMG Interpretation	ClinVar Interpretation	ClinVar ID	References
1	1	c.100G > A	p.Glu34Lys	rs371030381	0.00001626	VUCS	VUS	536202	
1	1	c.142G > A	p.Glu48Lys	rs1278890129	0.00002190	VUCS	P/VUS	440707	
1	1	c.151G > C	p.Gly51Arg			VUCS			
1	2	c.382G > A	p.Gly128Ser	rs766314770	0.00003978	VUCS			
1	3	c.411G > T	p.Leu137Phe			VUCS			
1	3	c.520C > T	p.Pro174Ser	rs533273863	0.00007089	VUCS	VUS	496561	
1	3	c.523 + 2T > G				LP			
1	5	c.709C > T	p.Arg237Trp	rs148195424	0.0006952	VUCS	VUS/LB	265933	
1	5	c.751C > T	p.Arg251Cys	rs778900671	0.00002009	VUCS			
1	7	c.1046G > A	p.Gly349Glu			VUCS			
1	7	c.1069C > T	p.Arg357Cys	rs148562777	0.0001450	LP	VUS	575758	
1	7	c.1070G > A	p.Arg357His	rs370507566	0.00003978	VUCS	VUS	403288	
1	7	c.1120G > A	p.Asp374Asn	rs137852912	0.00007079	LP			
3	9	c.1399C > G	p.Pro467Ala	rs772677312	0.00002829	LP	LP/VUS	265944	
1	9	c.1483C > T	p.Arg495Trp	rs758999339	0.00001607	VUCS			
2	9	c.1487G > A	p.Arg496Gln	rs139669564	0.0002363	VUCS	VUS/LB	438338	
1	10	c.1621C > T	p.Pro541Ser	rs369996097	0.00001056	VUCS			
0	11	c.1834G > A	p.Glu612Lys			VUCS			[32]
1	12	c.1903T > C	p.Cys635Arg			VUCS			
1	12	c.1939G > C	p.Ala647Pro			VUCS			
1	12	c.2002A > G	p.Ser668Gly	rs775077080	0.00004790	VUCS	VUS	297707	
1	12	c.2004C > A	p.Ser668Arg	rs762298323	0.00002397	VUCS	VUS	403291	
1		c.*415G > A				VUCS			[32]

^1^ Only for variants found in this study a number of index patients is given. Variants from systematic review are labelled with “0”.

**Table A3:** List of the *APOB* variants described in Russian patients.

Number of Index Patients ^1^	Exon	DNA Change	Protein Change	dbSNP ID	gnomAD MAF (v. 2.1.1)	ACMG Interpretation	ClinVar Interpretation	Clinvar ID	References
1	26	c.4298C > T	p.Ser1433Leu	rs200708197	7.583 × 10^5^	VUCS	VUS	630306	
1	26	c.4709T > C	p.Leu1570Ser			VUCS			
1	26	c.7057C > T	p.Gln2353Ter			LP			
1	26	c.10385A > G	p.Tyr3462Cys			VUCS			
1	26	c.10579C > T	p.Arg3527Trp	rs144467873	0.0001595	LP	P/LP	40223	
23	26	c.10580G > A	p.Arg3527Gln	rs5742904	0.0002942	LP	P/LP	17890	[9,35,36]
1	26	c.10672C > T	p.Arg3558Cys			VUCS			
3	26	c.10708C > T	p.His3570Tyr	rs201736972		VUCS			
1	26	c.11477C > T	p.Thr3826Met	rs61744153	0.001592	VUCS	LP/VUS/LB/B	237735	
1	28	c.11911G > A	p.Glu3971Lys			VUCS			
0	28	c.12005C > T	p.Ala4002Val	rs369364335	1.195 × 10^5^	VUCS	VUS	898076	[32]
1	29	c.12739C > T	p.Gln4247Ter	rs907126709		LP	VUS	544074	
1	29	c.13175G > A	p.Ser4392Asn			VUCS			
1	29	c.13480_13482del	p.Gln4494del	rs756545438		VUCS	LP/VUS	265896	

^1^ Only for variants found in this study a number of index patients is given. Variants from systematic review are labelled with “0”.

**Table A4:** Patients with multiple variants.

Patient Numbers	Phenotype	Variant 1 (Gene/Variant/Zygosity)	Variant 2 (Gene/Variant/Zygosity)	Cis/Trans Position (Evidence)
423	HoFH	*LDLR*:p.Gly592Glu (het)	*LDLR*:p.Glu418Ter (het)	Trans (genetic test of relatives)
474	Severe HetFH	*LDLR*:p.Gly592Glu (het)	*LDLR*:p.Ala771Glufs*9 (het)	Trans (long read sequencing)
166	HoFH	*LDLR*:c.941-3C > G (het)	*LDLR*:p.Cys329Tyr (het)	Trans (genetic test of relatives)
722	HoFH	*LDLR*:c.940 + 3_940 + 6del (het)	*LDLR*:p.Arg416Trp (het)	Unknown
668	HoFH	*LDLR*:p.Cys329Tyr (het)	*LDLR*:p.Gly592Glu (het)	Trans (genetic test of relatives)
675	HoFH	*LDLR*:p.Trp577Arg (hom)		
355	HoFH	*LDLR*:p.Ile441Asn (het)	*LDLR*:p.Ile792LeufsTer137 (het)	Unknown
687	HetFH	*LDLR*:p.Leu401His (het)	*PCSK9*:p.Arg357Cys (het)	
211	Severe HetFH	*LDLR*:p.Cys329Tyr (het)	*LDLR*:p.Gly592Glu (het)	Cis (genetic test of relatives)
336	HetFH	*LDLR*:p.Lys390Glu (het)	*PCSK9*:p.Glu34Lys (het)	
R-6	HetFH	*LDLR*:p.Val429Met (het)	*APOB*:p.Glu3971Lys (het)	
R-35	HetFH	*LDLR*:p.Gly592Glu (het)	*APOB*:p.Arg3527Gln (het)	
R-83	HetFH	*LDLR*:p.Gly592Glu (het)	*APOB*:p.Ser4392Asn (het) + *PCSK9*:p.Gly51Arg (het)	
R-115	HetFH	*APOB*:p.Arg3527Gln (het)	*PCSK9*:p.Pro174Ser (het)	
969	HetFH	*APOB*:p.Arg3527Gln (het)	*APOB*:p.Gln4494del (het)	Unknown
